# Effects of different designs of orthodontic clear aligners on the maxillary central incisors in the tooth extraction cases: a biomechanical study

**DOI:** 10.1186/s12903-023-03106-8

**Published:** 2023-06-22

**Authors:** Xuehuan Meng, Chunjuan Wang, Wenjie Xu, Rui Wang, Leilei Zheng, Chao Wang, Raffaella Aversa, Yubo Fan

**Affiliations:** 1 Stomatological Hospital of Chongqing Medical University, No.426 Songshibei Road, Yubei District, Chongqing, 401147 China; 2 Chongqing Key Laboratory of Oral Diseases and Biomedical Sciences, No.426 Songshibei Road, Yubei District, Chongqing, 401147 China; 3 Chongqing Municipal Key Laboratory of Oral Biomedical Engineering of Higher Education, No.426 Songshibei Road, Yubei District, Chongqing, 401147 China; 4 Key Laboratory of Biomechanics and Mechanobiology, Ministry of Education, Beijing Advanced Innovation Center for Biomedical Engineering, School of Biological Science and Medical Engineering, School of Engineering Medicine, Beihang University, No.37, Xueyuan Road, Beijing, 100083 China; 5 Advanced Material Lab, University of Campania, Luigi Vanvitelli, Caserta, Italy

**Keywords:** Clear aligner, Extraction case, Power ridge, Finite Element Method, Biomechanics

## Abstract

**Background:**

Controlling the 3D movement of central incisors during tooth extraction cases with clear aligners is important but challenging in invisible orthodontic treatment. This study aimed to explore the biomechanical effects of central incisors in tooth extraction cases with clear aligners under different power ridge design schemes and propose appropriate advice for orthodontic clinic.

**Methods:**

A series of Finite Element models was constructed to simulate anterior teeth retraction or no retraction with different power ridge designs. These models all consisted of maxillary dentition with extracted first premolars, alveolar bone, periodontal ligaments and clear aligner. And the biomechanical effects were analysed and compared in each model.

**Results:**

For the model of anterior teeth retraction without power ridge and for the model of anterior teeth no retraction with a single power ridge, the central incisors exhibited crown lingual inclination and relative extrusion. For the model of anterior teeth no retraction with double power ridges, the central incisors tended to have crown labial inclination and relative intrusion. For the model of anterior tooth retraction with double power ridges, the central incisors exhibited a similar trend to the first kind of model, but as the depth of the power ridge increased, there was a gradual decrease in crown retraction value and an increase in crown extrusion value. The simulated results showed that von-Mises stress concentration was observed in the cervical and apical regions of the periodontal ligaments of the central incisors. The clear aligner connection areas of adjacent teeth and power ridge areas also exhibited von-Mises stress concentration and the addition of power ridge caused the clear aligner to spread out on the labial and lingual sides.

**Conclusions:**

The central incisors are prone to losing torque and extruding in tooth extraction cases. Double power ridges have a certain root torque effect when there are no auxiliary designs, but they still cannot rescue tooth inclination during tooth retraction period. For tooth translation, it may be a better clinical procedure to change the one-step aligner design to two-step process: tilting retraction and root control.

**Supplementary Information:**

The online version contains supplementary material available at 10.1186/s12903-023-03106-8.

## Background

With the increasing demand for cosmetic treatments, traditional fixed appliances can no longer meet the aesthetic needs of orthodontic patients. The concept of modern orthodontics not only requires improving the patient’s aesthetic appearance after orthodontic treatment, but also aims to achieve aesthetic standards in the process of treatment. In early years, lingual appliances were popular among orthodontic patients due to their excellent aesthetic effects [1]. Clear aligners have emerged with the development of computer science and technology. Of all appliances, clear aligners are the most beautiful, comfortable, convenient, and beneficial for oral hygiene. Additionally, using clear aligner treatment greatly reduces the amount of time spent in the doctor’s chair [2–6]. Therefore, the clear aligners have quickly become the favourite of the orthodontic industry.

Clear aligners are a series of transparent, elastic, movable correction devices designed and manufactured by digital technology. Each step of the aligners differs slightly from the current position of the teeth. The teeth are gradually moved to the target position by the elastic restoring force generated when wearing the aligner on the crown, and each-step aligner is designed to move teeth by 0.1-0.3 mm. Patients are required to wear each-step aligner for approximately two weeks, and for at least 20 h per day [7, 8].

Of course, clear aligners also have some limitations. For example, the lack of specific force application points can lead to insufficient force on teeth, and materials are prone to aging in the oral cavity. Therefore, tooth movement efficiency is not always as good as predicted. Moreover, some patients may require multiple stages of intermediate refinement or additional aligners, and even need to switch to fixed orthodontics before completing the treatment [9–11]. However, because the advantages of clear aligners far outweigh the disadvantages, invisible orthodontics continues to thrive and improve.

Early studies have shown that clear aligners perform well in distal movement of maxillary molars, alignment of anterior teeth, and expansion of the anterior arch. However, they are not effective in extruding anterior teeth, rotating round teeth or translating teeth [11–16]. With the rapid development of materials science and manufacturing technology, invisible orthodontic technology has been continuously improved, expanding its indications from initial mild to moderate malocclusion to complex malocclusion cases that include tooth extraction [17–19].

In orthodontic clinic, crowded dentition and protruding profile are common complaints that often require tooth extraction. En-mass retraction of anterior teeth is a commonly used method for closing the extraction space [20]. Whether in fixed orthodontics or invisible orthodontics, the accuracy of 3D control of upper incisors is crucial as the position and angle of anterior teeth are closely related to final aesthetics [21]. For invisible orthodontics, 3D control is more difficult because moving the teeth with clear aligner alone is more likely to induce “the roller coaster effects”, such as torque loss and overbite deepening of anterior teeth. This can lead to unsatisfactory aesthetic results when orthodontic treatment is finished [22–24]. For example, Li et al. compared the tooth movement effects of invisible orthodontics and fixed orthodontics, and found that the control effect of fixed orthodontics was better than that of invisible orthodontics [25]. Furthermore, Dai et al. investigated the actual and predicted movements of central incisors in cases where first premolars were extracted, and discovered that compared to the predictions, the lingual inclination of the central incisor was greater while extrusion was more and retraction was less [18, 19].

A ‘power ridge’ has been designed on the clear aligners corresponding to the incisors in invisible orthodontics, especially in tooth extraction cases, for better control the torque of incisors. Some scholars have confirmed that adding a power ridge to the labial gingival area of the upper central incisor has a better torque control effect than using a horizontal ellipsoid attachment [12, 26]. Cheng et al.’s study shows that clear aligners of different thicknesses need to be combined with different height power ridges to achieve bodily movement of central incisors, 0.5 mm-thick aligner better accompanied with a power ridge of 0.7 mm while 0.75 mm-thick aligner better accompanied with a power ridge of 0.25 mm [24]. However, it is unclear how the addition of power ridges to the clear aligner system will affect its overall performance and whether different designs of power ridges will have varying biomechanical effects on the incisors.

The aim of this study was to investigate the biomechanical effects of invisible orthodontic extraction cases and the influence of different power ridge designs on tooth movement, as well as to explore the appropriate design of 3D control scheme of anterior teeth in tooth extraction cases. Due to the complexity of the orthodontic force system, in vivo research is not feasible [27–29]. In this study, the stress, strain and displacement of the orthodontic loading model was calculated using 3D Finite Element Method, which has strong repeatability [30–32].

## Methods

### Original data and designs

**Fig. 1:**
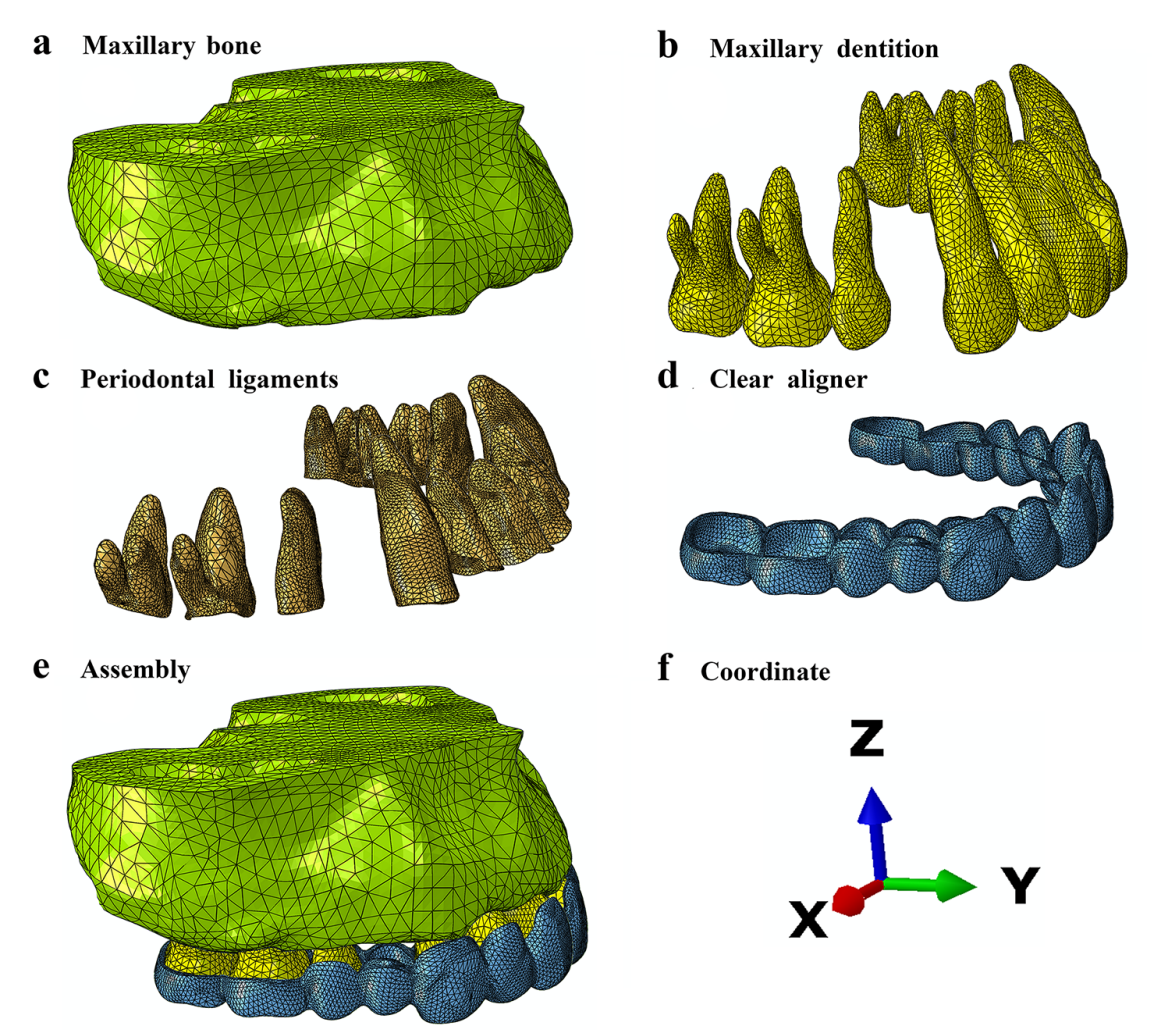
(**a**) The model of maxillary bone; (**b**) The model of maxillary dentition; (**c**) The model of maxillary periodontal ligaments; (**d**) The model of clear aligner; (**e**) The model assembly; (**f**) The global coordinate system

An adult orthodontic patient (female, 26 years old, from Department of Orthodontics, Stomatological Hospital of Chongqing Medical University) with a protrude profile was selected, her teeth, mucosa and periodontal tissue were healthy, and she had no temporomandibular joint or other lesions. After the maxillary bilateral first premolars were extracted and the maxillary dentition was aligned, the dental cone-beam computed tomography scan of the patient was obtained and saved. The data of right maxillary alveolar bone and dentition were input into Mimics19.0 software (Materialise, Leuven, Belgium) to construct the initial model. After smoothed and mirrored by GeomagicStudio2015 software (Geomagic, Morrisville, NC, USA) and SolidWorks2016 software (Dassault Systems, Concord, MA, USA), the bilaterally symmetrical maxillary bone (Fig. 1a) and maxillary dentition (Fig. 1b) were reconstructed. Periodontal ligaments (Fig. 1c) with an average thickness of 0.2 mm [33] were constructed along the root anatomy while the aligner (Fig. 1d) with an average thickness of 0.75 mm [34] was constructed along the crown anatomy (Supplementary Methods). Therefore, each model was consisted of maxillary dentition with extracted first premolars, alveolar bone, periodontal ligaments and clear aligner. All components were combined to generate an assembly (Fig. 1e).

**Fig. 2:**
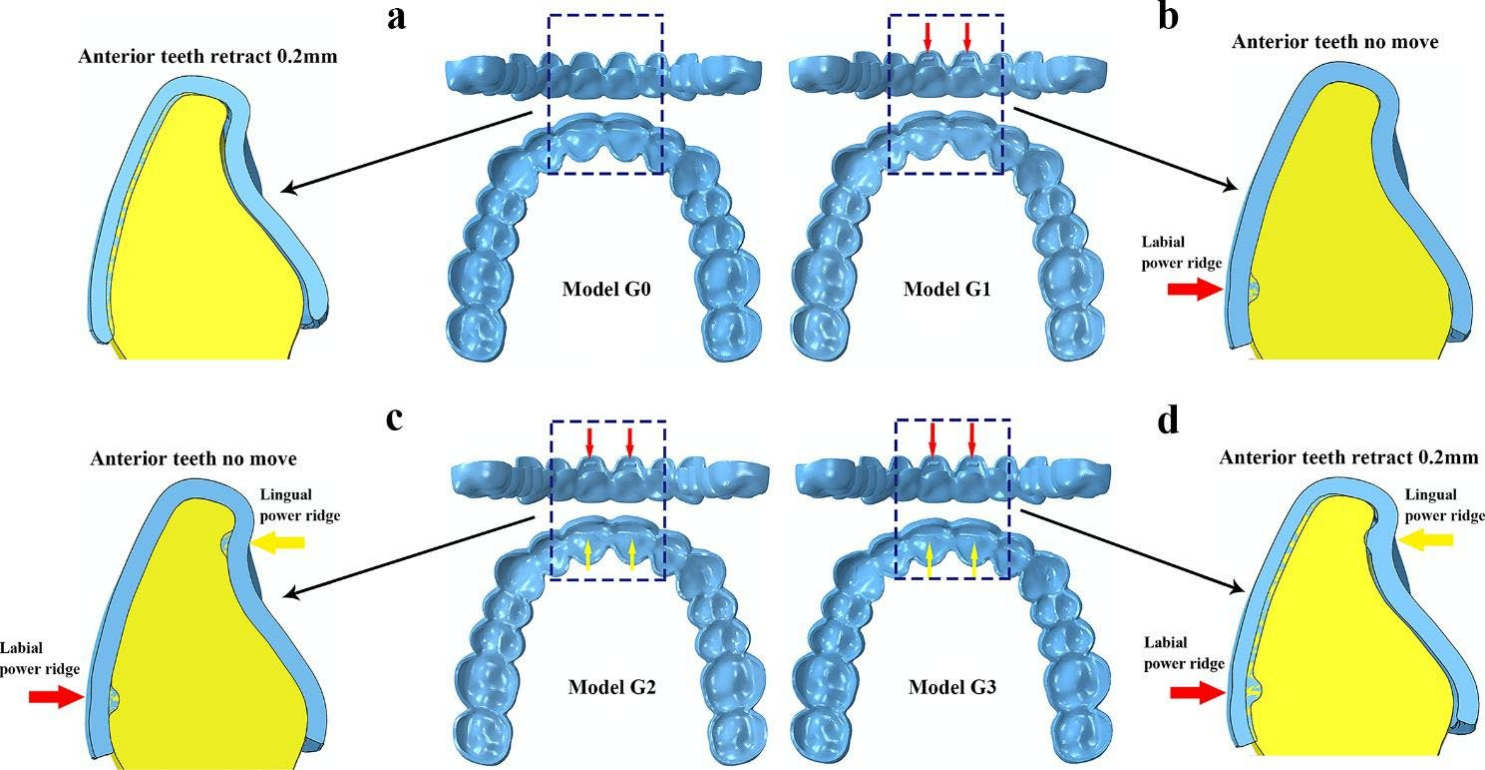
(**a**) The clear aligner design of Model G0; (**b**) The clear aligner design of Model G1; (**c**) The clear aligner design of Model G2; (**d**) The clear aligner design of Model G3

For our four major groups, all were each-step aligner designs and only the clear aligner differ among each group. The aligner models were designed as follows (Fig. 2):

- Model G0 (Fig. 2a), the clear aligner was constructed based on the crown position (anterior teeth (from 13 to 23) en-masse retracted 0.2 mm) without power ridge. This simulated the situation that the anterior teeth were retracted by the aligner alone without any auxiliary accessories. The goal of this design was to verify whether using clear aligner alone can achieve the effect of translation of anterior teeth.
- Model G1 (Fig. 2b), the clear aligner was constructed based on the original crown position, which featured power ridge at central incisor labial side. This simulated the situation that a single power ridge acted on the central incisor without tooth movement designs. The goal of this design was to verify whether a single power ridge can achieve the effect of root control.
- Model G2 (Fig. 2c), the clear aligner was constructed based on the original crown position, which featured power ridges at central incisor both labial side and lingual side. This simulated the situation that double power ridges acted on the central incisor without tooth movement designs. The goal of this design was to verify whether double power ridges can achieve the effect of root control.
- Model G3 (Fig. 2d), the clear aligner was constructed based on the crown position (anterior teeth (from 13 to 23) en-masse retracted 0.2 mm) with power ridges at central incisor both labial side and lingual side. This simulated the comprehensive situation of retracting anterior teeth with double power ridges. The goal of this design was to verify whether adding double power ridges to the clear aligner has better translation effect during anterior teeth retraction period compared with the same situation without power ridge.

The power ridges were located at gingival third of labial side and the incisal third of lingual side, which was rectangular with 3 mm long and 1 mm wide determined according to clinical measurement. For G1-G3, four kinds of power ridge depths were designed including 0.2 mm, 0.3 mm, 0.4 and 0.5 mm (written as G1-0.2, G1-0.3, G1-0.4, G1-0.5, etc.). The clear aligner models were built in the anterior teeth retracted positions in G0 and G3, but in the original tooth positions in G1 and G2. For ease of description, the positions built clear aligners were collectively called the target positions of tooth movement. All models were imported into the Finite Element software Abaqus6.14 (Simulia, France) for calculations.

### Material properties and meshing

The material properties (Table 1) were established according to previous studies [35–38]. The clear aligner was set large deformation, and the adaptive mesh division method provided by Abaqus6.14 software was used to freely divide the mesh. The mesh sizes for tooth, alveolar bone, periodontal ligament and clear aligner were 1.0 mm, 2.0 mm, 0.5 and 0.6 mm, respectively. Mesh sizes were defined after the preliminary experiment results converged (Fig. S1). The numbers of nodes and elements are shown in Table 2.

**Table 1:** Material properties

Components	Young’s modulus (MPa)	Poisson’s ratio
Tooth	1.86 × 10^4^	0.31
Alveolar bone	1.37 × 10^4^	0.30
Periodontal ligament	0.68	0.49
Clear aligner	816.31	0.30

**Table 2:** Numbers of nodes and elements after meshing

Components	Nodes	Elements
Tooth	136,050	87,830
Alveolar bone	97,212	63,506
Periodontal ligament	165,086	81,882
Clear aligner (G0)	177,012	101,262
Clear aligner (G1-0.2)	188,598	107,878
Clear aligner (G1-0.3)	190,247	108,680
Clear aligner (G1-0.4)	191,984	109,730
Clear aligner (G1-0.5)	193,760	110,610
Clear aligner (G2-0.2)	193,994	111,078
Clear aligner (G2-0.3)	195,926	112,238
Clear aligner (G2-0.4)	197,698	113,382
Clear aligner (G2-0.5)	199,946	114,398
Clear aligner (G3-0.2)	194,022	111,226
Clear aligner (G3-0.3)	195,990	112,690
Clear aligner (G3-0.4)	197,946	113,714
Clear aligner (G3-0.5)	199,994	114,674

### Boundary constraints and contact conditions

The base of the alveolar bone was set fixed constraint, and the whole model was set symmetry constraint with the mid-sagittal plane as the symmetry axis. The interfaces between the tooth and periodontal ligament and between the periodontal ligament and alveolar bone were set bonded contact. A nonlinear surface-to-surface contact relationship between the outer surface of the crown and the inner surface of the clear aligner was set, with a friction coefficient of µ = 0.2 [39], no constraints or loads were applied to the tooth or the clear aligner (Fig. S2).

**Fig. 3:**
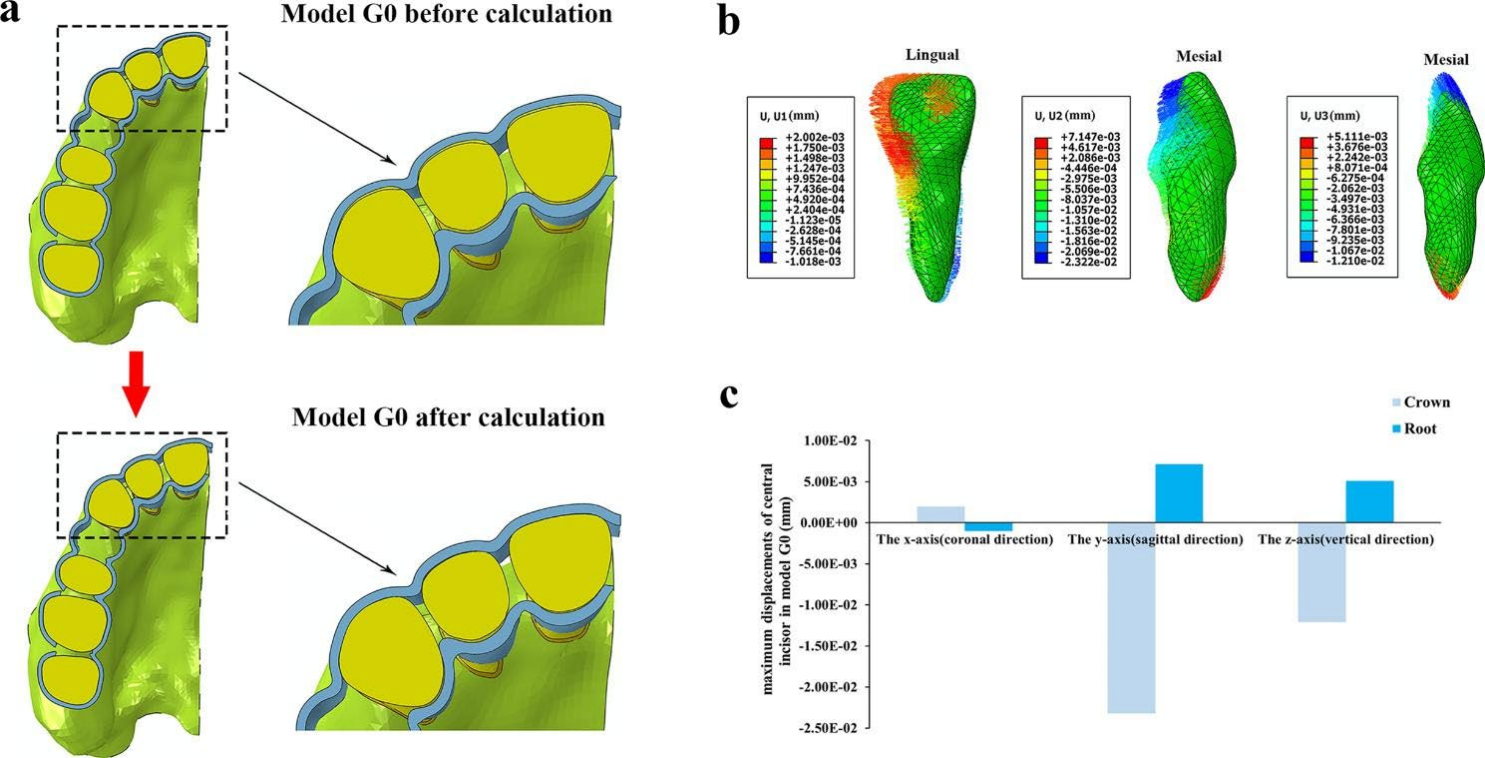
(**a**) Model G0 before and after calculation; (**b**) The displacement pattern of the right central incisor in model G0; (**c**) The maximum displacements of the right central incisor in model G0

***Activation of tooth movement calculation.*** The crowns and clear aligners overlapped each other in the anterior tooth regions in the assembly models (Fig. 2), for the clear aligners were built at the target positions of tooth movement while the tooth models were at original positions in all assembly models. Although the aligners of G1 and G2 were constructed based on the original tooth positions, the power ridge is the ridge that bulge inward based on the outer surface of the crown, so there was still overlap between the aligners and the central incisor crowns. Our calculation simulated the process of wearing clear aligners onto the crowns (Fig. 3a), which was similar to contact interference assembly in mechanical engineering [40].

Interference assembly is initial interference between the two assembly models, as the object develops stress deformation, it is tightly assembled together. In our study, the surface-to-surface contact pairs algorithm was adopted which composed of a master surface and a slave surface (Fig. S2). The crown surface with high stiffness was set as the master surface, and clear aligner was set as the slave surface. All contact directions were along the normal direction of the master surface, and the nodes on the slave surface could not penetrate the master surface, but the nodes on the master surface could penetrate the slave surface. In order to resolve the interference between the clear aligner and the crown, the automatic shrink fit algorithm [41–43] which gradually removed slave node over-closure during the analysis step was applied to resolve the interference, the tooth moved towards the target position of the clear aligner also with clear aligner moved towards the tooth to change the mismatch into match, thus activated the calculation process of invisible orthodontics automatically. The interference resulted in stresses and strains in the model as over-closure was resolved (Figs. S3 and S4).

### Coordinate system setting

The x-axis, y-axis and z-axis of the 3D coordinate represent the coronal direction, sagittal direction and vertical direction, respectively (Fig. 1f); and the distal, labial, and root directions of the right central incisor indicate the positive directions of the x-axis, y-axis and z-axis, respectively.

### Calculation and analysis

The nonlinear iterative calculations (Supplementary Methods, Figs. S3 and S4) were carried out by Abaqus, and the items analysed included:

- initial displacement patterns of central incisors.
- von-Mises stress distributions of periodontal ligaments of central incisors.
- deformation trends and von-Mises stress distributions of clear aligners.

## Results

**Fig. 4:**
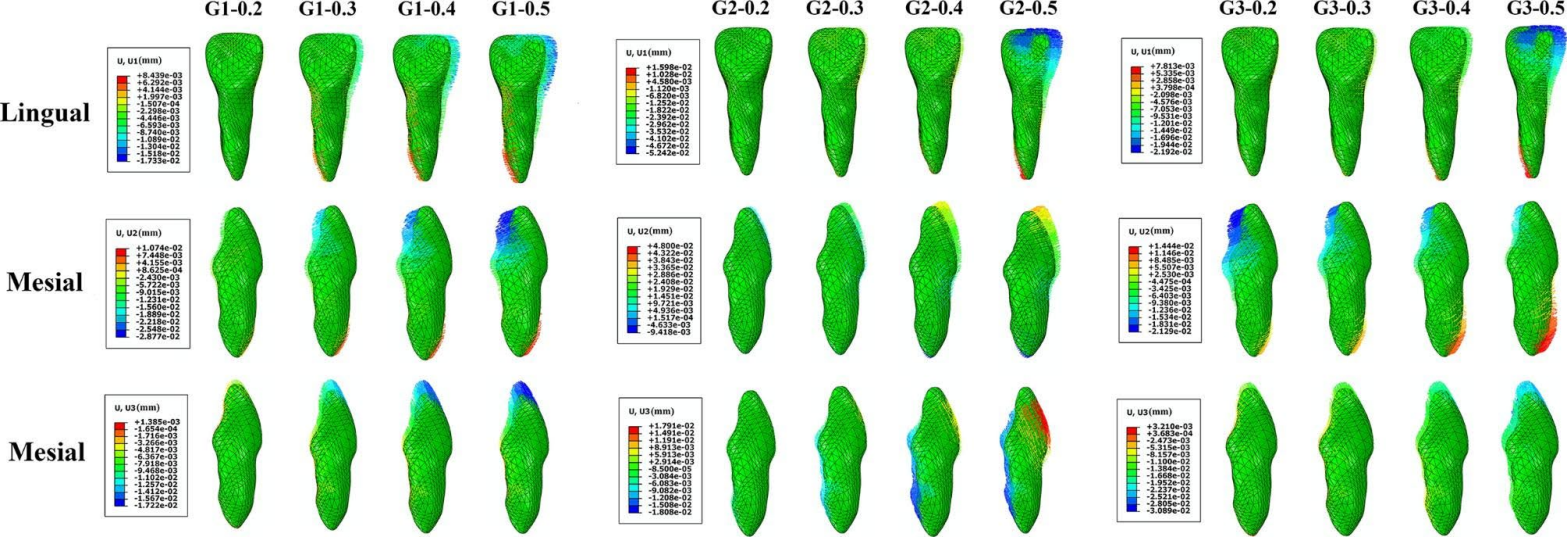
The displacement patterns of the right central incisors in model G1-G3

**Fig. 5:**
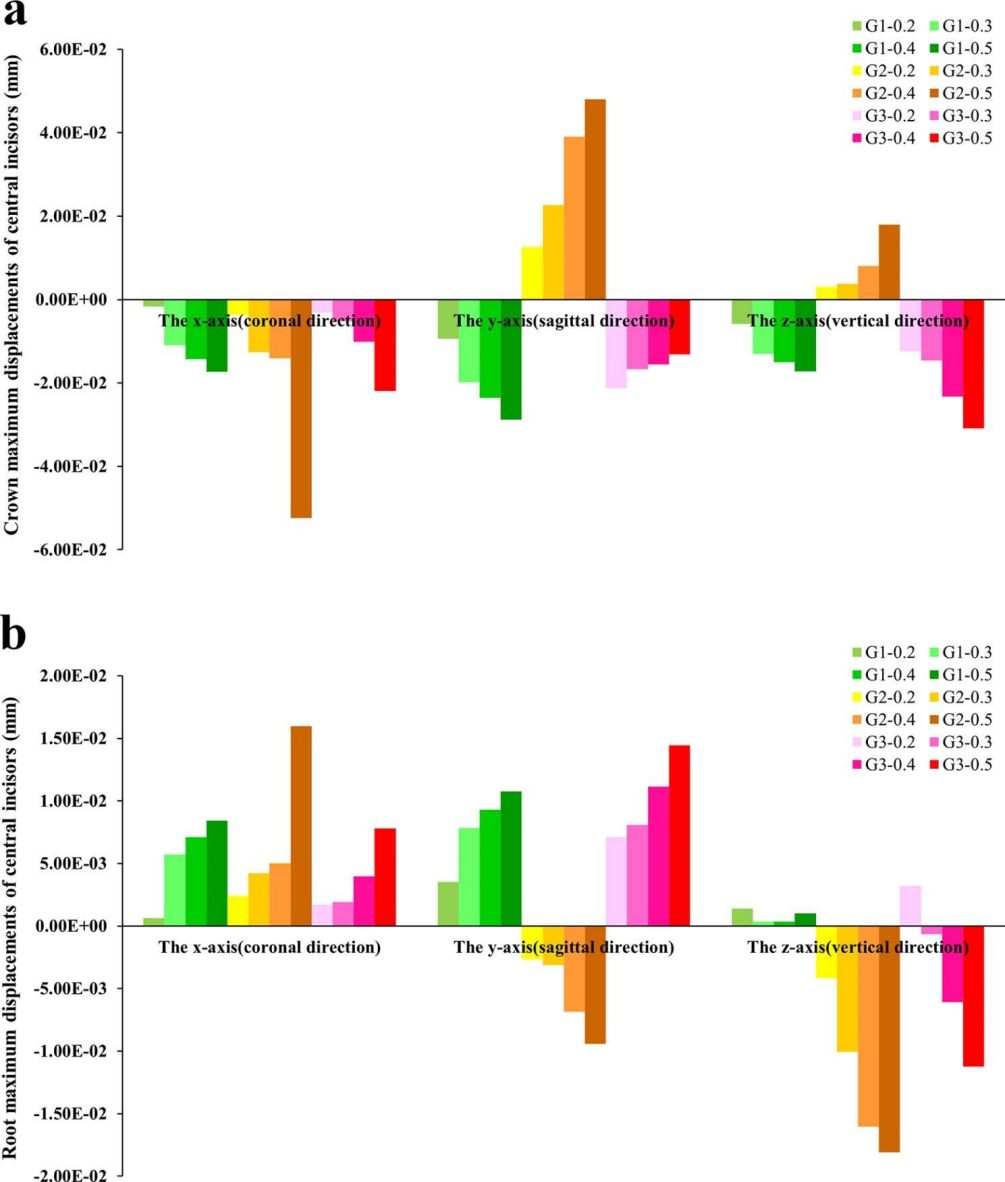
(**a**) The crown maximum displacements of the right central incisors in model G1-G3; (**b**) The root maximum displacements of the right central incisors in model G1-G3

For the displacement patterns of central incisors, the crowns and roots exhibited movements in the opposite directions in each axis except the z-axis of G3, the results are shown in Figs. 3, 4 and 5.

- G0: The central incisor showed distal crown tipping, lingual crown tipping and crown relative extrusion (Fig. 3b and c).
- G1: The displacement patterns of all subgroups were similar; as the depth of the power ridge increased, the displacement values of both crowns and roots increased exclude the root displacement of G1-0.2. Each subgroup showed mesial crown inclination. Besides, lingual crown tipping with crown mesial lingual torsion and crown relative extrusion were observed (Figs. 4 and 5).
- G2: The displacement patterns of all subgroups were also analogous; as the power ridge depth increased, the displacements of central incisors also increased. It showed mesial crown inclination and labial crown tipping. Besides, crown relative intrusion was observed (Figs. 4 and 5).
- G3: All subgroups showed mesial crown inclination which similar to G1 and G2. In the sagittal direction, all subgroups showed lingual crown tipping; unlike G1 and G2, as the depth of the power ridge increased, the displacements of crowns gradually decreased but the displacements of roots gradually increased. G3-0.2 showed crown relative extrusion and root relative intrusion, whereas the other subgroups showed absolute tooth extrusion (Figs. 4 and 5).

**Fig. 6:**
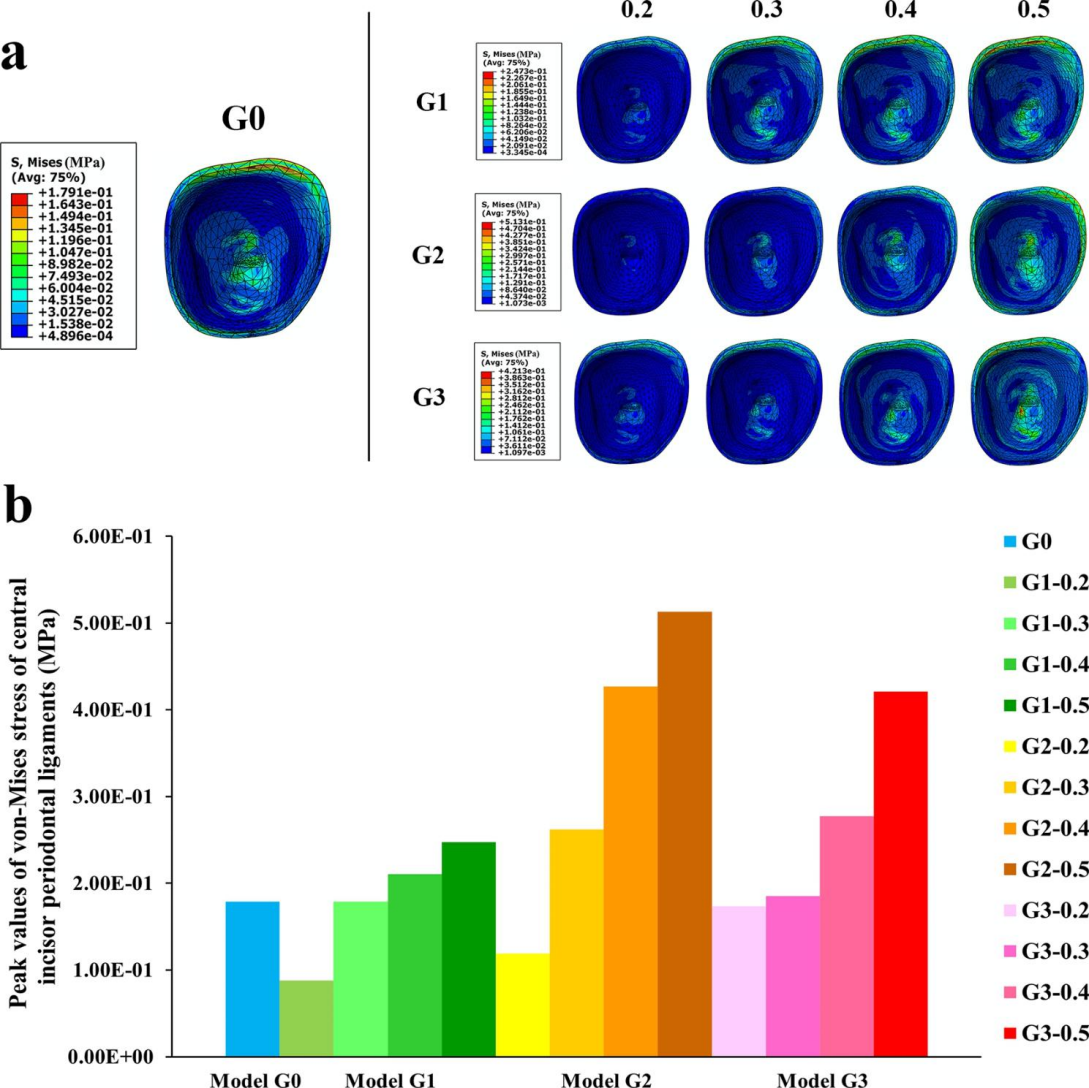
(**a**) The von-Mises stress distribution of the right central incisor periodontal ligament in each model; (**b**) The peak value of von-Mises stress of the right central incisor periodontal ligament in each model

The cervical areas and root apexes of periodontal ligaments of all central incisors revealed von-Mises stress concentrations (Fig. 6a), which were consistent with tooth displacement patterns. For G1-G3, as the depth of the power ridge increased, the peak values of von-Mises stress increased gradually (Fig. 6b).

**Fig. 7:**
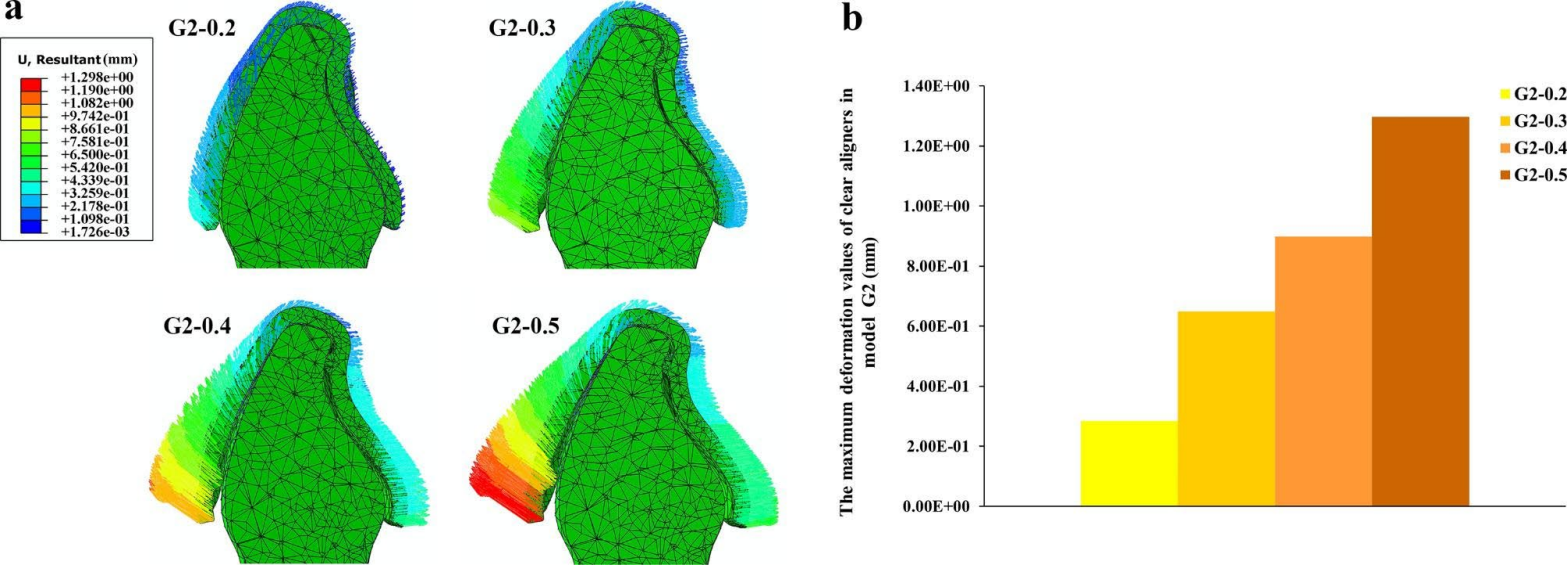
(**a**) The deformation trends of the clear aligners in model G2; (**b**) The maximum deformation values of the clear aligners in model G2

The addition of power ridges caused the clear aligner to spread out on the labial and lingual sides in each model (Fig. 7a). As the power ridge depth increased, the spreading degree gradually increased (Fig. 7b), but the maximum deformation values were all located at the labial cervical areas of clear aligners (Fig. 7a).

**Fig. 8:**
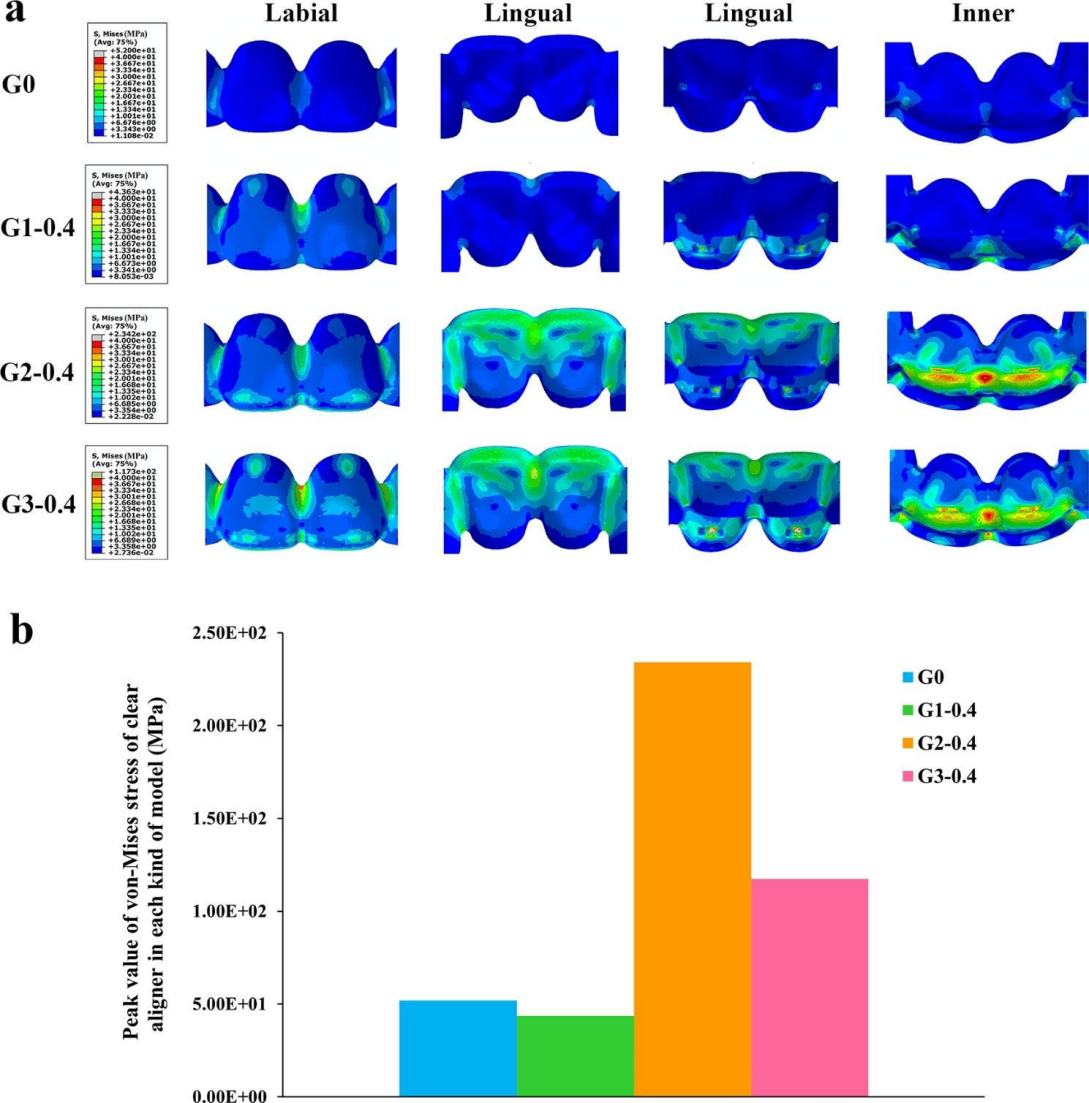
(**a**) The von-Mises stress distribution of the clear aligner in each kind of model; (**b**) The peak value of von-Mises stress of the clear aligner in each kind of model

The connection areas between adjacent teeth appeared von-Mises stress concentration in each group; besides, von-Mises stress concentration also showed on the power ridge areas in G1 and the power ridge areas and the incisal edges in G2 and G3 (Fig. 8a). The clear aligners with double power ridges exhibited lager peak values of von-Mises stress than the aligners without power ridge or with a single power ridge (Fig. 8b).

## Discussion

The efficiency of tooth movement in invisible orthodontics has been the focus of many previous studies [12–16], but its biomechanics are still not very clear [8, 19, 44]. In recent years, several Finite Element studies have attempted to explore the biomechanical effects of invisible orthodontics. However, those models were mainly based on one or several teeth and applied direct force to the teeth or displacement load on clear aligners to simulate the invisible orthodontic process. These approaches differ significantly from real clinical situations [39, 44–46]. Additionally, there is limited research on the use of invisible treatment for cases involving tooth extraction. In orthodontic clinic, the clear aligner exerts orthodontic force to correct malocclusion by deforming due to misalignment with the dentition position. This process is similar to the theory of interference assembly in mechanical engineering [40]. Interference assembly refers to the initial interference between two assembly models, which become tightly assembled together as stress deformation occurs. This process is similar to wearing clear aligners on teeth. To simulate an interference fit, a special algorithm called automatic shrink fit [41] was used in this study. Interferometers were gradually eliminated during analysis and calculation, and deformation stresses were generated. Therefore, our simulation was reasonable and highly consistent with clinical practice.

In extraction cases, sagittal and vertical controls of central incisors are crucial, especially sagittal control. Our results indicate that the central incisor exhibited a loss of torque and tendency towards deepening overbite in G0 (Fig. 3b), which is consistent with the findings of Dai et al. [18, 19, 24]. In G1, a counterclockwise (mesial view) movement pattern in the sagittal direction was observed, along with an increase in overbite in the vertical direction (Fig. 4). However, in G2, the central incisors exhibited a clockwise (mesial view) movement pattern in the sagittal direction and there was a decrease in overbite in the vertical direction (Fig. 4). In the absence of other designs, single power ridge has no effect on lingual root torque or opening occlusion. However, double power ridges appear to have an effect. Therefore, we designed G3 with the expectation of achieving better 3D control of central incisors and achieving the desired effect of tooth translation.

Unfortunately, the result was not ideal. In orthodontic clinic, the absolute value of the crown-root displacement ratio in the sagittal direction can be used to evaluate the degree of tooth translation. When the direction of crown and root displacement is opposite, the larger the absolute value of crown-root displacement ratio, the more likely teeth are to move bodily. From the results, we can see that although double power ridges have a certain torque control effect in situ, adding them during anterior teeth retraction cannot achieve tooth translation and may even significantly sacrifice the amount of retraction displacement. As the power ridges deepen, the teeth become increasingly oblique, resulting in less displacement of crown retraction and greater labial inclination of the root, which was not desirable (Figs. 3, 4 and 5; Table 3). Compared with G0, even though the root labial movement of G3-0.2 reduced, the displacement of crown retraction also reduced, the absolute value of crown-root displacement ratio was still smaller than G0 (Figs. 3c and 5; Table 3), so the teeth tended to move more obliquely than G0. In the vertical direction, the power ridges did not reduce the overbite but deepened it (Figs. 3c and 5a). On the other hand, we compared the maximum stresses of periodontal ligaments in both G0 and G3, from which we can see that with the depth of power ridges increased, the maximum von-Mises stress of periodontal ligament also increased, only the stress of G3-0.2 was smaller than G0 (Fig. 6b). Therefore, according to the absolute values of crown-root displacement ratio in sagittal direction and the stresses of periodontal ligaments, it can be inferred that the addition of power ridges during the anterior teeth retraction period cannot achieve the desired effect.

**Table 3:** The absolute values of crown-root maximum displacement ratio in sagittal direction in G0 and G3 and peak values of von-Mises stress of periodontal ligaments in G0 and G3

Groups	Crown maximum displacements in sagittal direction (mm)	Root maximum displacements in sagittal direction (mm)	Absolute values of crown-root maximum displacement ratio in sagittal direction	Peak values of von-Mises stress of periodontal ligaments (MPa)
G0	-2.32E-02	7.15E-03	3.25	1.79E-01
G3-0.2	-2.13E-02	7.09E-03	3.00	1.73E-01
G3-0.3	-1.67E-02	8.07E-03	2.07	1.85E-01
G3-0.4	-1.56E-02	1.12E-02	1.40	2.78E-01
G3-0.5	-1.31E-02	1.44E-02	0.91	4.21E-01

From the results of this study, there were no significant differences in tooth displacement patterns between different power ridge depth designs (Fig. 4), but increasing the depth resulted in higher tooth displacement values (Fig. 5). In addition, the results of G0 showed that retracting anterior teeth with clear aligners alone could lead to tooth tipping (Fig. 3b). G2 and G3 demonstrated that double power ridges controlled lingual root torque in situ, but adding them during the retraction period had an adverse effect (Fig. 4). This indicates that the invisible orthodontic system is a very complex mechanical system, and its comprehensive effects are not simply a superposition of individual outcomes when it comes to tooth translation. When tooth translation cannot be achieved by one step, changing procedure to two steps of tilting retraction and root control may be a good choice.

Root resorption caused by excessive orthodontic force is also a concern in orthodontic treatment. Gay et al. measured root resorption during Invisalign clear aligner treatment and found that 81% of the 1083 teeth measured showed a reduction in root length compared to pre-treatment levels [47]. Another study analysed a total of 640 teeth for root length alteration in orthodontics, with 320 in fixed orthodontics and 320 in invisible orthodontics. The mean percentage value of external apical root resorption in the invisible orthodontics group was 5.13 ± 2.81%, which was significantly less than that of the fixed orthodontics group (6.97 ± 3.67%) [47]. In this study, all periodontal ligament apexes showed high levels of von-Mises stress concentration (Fig. 6a), indicating that the root apexes of the central incisors were subjected to significant stress. Additionally, as the depth of power ridges increased, so did the von-Mises stress, thereby the risk of root resorption could not be ignored. Therefore, orthodontists should control orthodontic force within an appropriate range by reducing step distance or power ridge depth to minimize root resorption.

The efficiency of tooth movement in invisible orthodontics largely depends on the fit between the clear aligner and crown [29, 48]. Our study found that the presence of a power ridge could lead to a lack of fit between the clear aligner and crown and increasing power ridge depth resulted in a greater degree of nonfit (Fig. 7), which may affect the wearing of the clear aligner, potentially impacting the effectiveness of orthodontic treatment. However, as mentioned before, increasing the depth of the power ridge could lead to increasing of tooth displacement if bone necrosis is not taken into consideration. Therefore, orthodontists should carefully consider both the positive and negative effects in order to create a comprehensive design.

In addition, each simulated model showed von-Mises stress concentration in the joint areas of adjacent teeth in the anterior part (Fig. 8a). It is evident that these areas are particularly susceptible to fracture or plastic deformation of clear aligners due to stress concentration. Therefore, it is necessary to reinforce the connection areas between adjacent teeth or improve the mechanical properties of materials used in these areas to reduce the risk of breakage.

Previous data shows that relying clear aligners alone cannot solve all the malocclusion deformities [49, 50]. Attachments play a crucial role in the process of invisible orthodontics, enabling better orthodontic force transmission to teeth, assisting tooth movement, and increasing aligners retention. The addition of attachments may help reduce the need for additional aligners, reduce the overall treatment duration, and provide more predictable treatment results than those with clear aligners alone [9]. Numerous studies have confirmed that the addition of attachments can significantly improve tooth movement efficiency compared to the absence of attachments [9, 51]. However, as the purpose of this study was to investigate the original biomechanical properties of the invisible orthodontic system, no attachment was designed in this study. Subsequent studies can try to add attachments, elasticity and other auxiliary devices, which may obtain different biomechanical effects.

As an efficient mathematical simulation method, Finite Element Method can reproduce complex geometric shapes and their physical characteristics on computers to evaluate their behavior after applying force, and is widely used in the dental field [52]. However, this method also has certain limitations which may lead to deviations between the simulation result and the actual one, such as the inability to truly simulate the oral environment, including temperature and saliva inside the mouth, as well as personalized bone morphology of different patients [53]. The simulation result largely depends on the parameter settings of the materials, and cannot simulate the dynamic changes of the dental system under long-term force loading and the clear aligner itself [24]. Therefore, although this simulated study helps us have intuitive understanding of the biomechanical effects of invisible orthodontics on anterior teeth retraction in tooth extraction cases, long-term and clinical trials are still needed to verify the results given in this study.

Another limitation that should be pointed out is that 13 complete maxillary models were constructed in this study, but only the central incisors were targeted designed and analysed. The force state of the whole dentitions were not thoroughly analysed. Therefore, future designs should include all teeth and analyse the biomechanical effects of the entire invisible orthodontic system to gain a deeper understanding.

## Conclusions

Within the limitations of this study, it can be concluded that in cases of invisible orthodontics involving first premolar extraction, there is a tendency for the central incisors to lose torque and extrude. Without other designs, a single power ridge does not have a lingual root torque effect, while double power ridges do. Tooth displacement patterns did not show significant differences among different power ridge depth designs, but increasing the depth of the power ridge could lead to an increase in tooth displacement value. When tooth translation cannot be achieved in one step, it is recommended to modify the clinical design of invisible orthodontics to include two steps: tilting retraction and root control.

## Electronic supplementary material

Below is the link to the electronic supplementary material.

Supplementary Methods and Supplementary Figs. 1-4

## Data Availability

The data used and analysed during the current study are available from the corresponding author on reasonable request.
