# Supplementary material for: Effects of different designs of orthodontic clear aligners on the maxillary central incisors in the tooth extraction cases: a biomechanical study
Source: BMC Oral Health. 2023 Jun 22;23:416. doi: 10.1186/s12903-023-03106-8 (PMC10288704; doi:10.1186/s12903-023-03106-8)
Supplement: Supplementary file 1 — Supplementary Methods and Supplementary Figs. 1-4 [file 12903_2023_3106_MOESM1_ESM.docx]

Supplementary Materials for

**Effects of different designs of orthodontic clear aligners on the maxillary central incisors in the tooth extraction cases: a biomechanical study**

**This file includes:**

Supplementary Methods

Supplementary Figs. 1-4

**Supplementary methods**

**Model construction**

The maxillary bone and dentition of the patient were scanned by Cone Beam Computed Tomography machine, and the medical image data was saved in DICOM format. After the DICOM data was imported into Mimics19.0 software, the initial 3D model of maxillary bone and maxillary dentition were obtained through medical image operations of threshold segmentation, region growing, smoothing and calculation of 3D and so on. The data of the right maxillary bone and maxillary dentition were then extracted and the models were subjected to filling, refinement and mirroring by Geomagic Studio 2015 software and Solidworks 2016 software, thereby bilaterally symmetrical complete maxillary bone and maxillary dentition models were constructed.

The maxillary dentition was imported into Geomagic Studio 2015 software, and each tooth was offset 0.2 mm along the normal direction, which was subjected to boolean subtraction with the original tooth and saved as IGES format. Then, the IGES format file and the maxillary bone model file were imported into Solidworks 2016 software, and the maxillary periodontal ligament models were obtained by Boolean intersection.

The maxillary anterior teeth were retracted 0.2 mm in the sagittal direction in Solidworks 2016 software, and the dentition at this time was marked as “M”. After “M” was imported into Geomagic Studio 2015 software, the gap between each two teeth were manual fused, and then the outer surface of the crown was extracted as the inner surface of the aligner, the aligner model was finally obtained after curve clipping, a shell of 0.75 mm and smoothing. For G1 and G2 groups, no tooth movement was designed so “M” meant the dentition in situ.

**Contact conditions**

***Contact pairs settings***

The contact between the clear aligner and the crown belongs to the flexible body-flexible body contact. Since the contact area is known, that is, the inner surface of the aligner is in contact with the crown surface, so the surface-to-surface contact pairs algorithm was adpoted in the contact type. The crown surface with high stiffness was set as the master surface, and clear aligner was set as the slave surface.

***Contact property definitions***

The interaction between the contact surfaces includes normal and tangential behavior. The normal behavior was set to "hard contact" relationship, it means the contact pressure that be transmitted between the contact surfaces could only be positive pressure and not limited. When the pressure on the contact surfaces becomes negative or zero, the two contact surfaces would be separated. Tangential behavior was set to penalty-based Coulomb friction model with friction coefficient of 0.2. The contact relative slip does not exceed a small ratio of the element's characteristic length, so the relative sliding between the contact surfaces adopted the "small sliding" formula.

***Interference load setting***

The loading of the clear aligner adopted the “interference fit” function of the Abaqus6.14 software. In the Finite Element Method, the contact problem follows the principle of impenetrability, that is, penetration between the master surface and the slave surface is not allowed. In order to resolve the interference between the clear aligner and the crown, the automatic shrink fit algorithm which gradually removed slave node over-closure during the analysis step was applied to resolve the penetration. The interferences resulted in stresses and strains in a model as over-closures are resolved.

**iterative calculations**

The nonlinear iterative calculations were carried out by Abaqus6.14 software, which is divided into several incremental iterative convergence in total. During the calculation process, the amount of interference decreased gradually, until the last incremental step, the amount of interference decreased to 0. This result was completely in line with the process of clear aligner wearing.

**Supplementary Figures**

**

**

**Fig. S1** The convergence test of preliminary experiment. When the number of the whole element reached 334480, which used the mesh size in this study, the calculation results of the maximum displacement of the central incisor began to converge

**
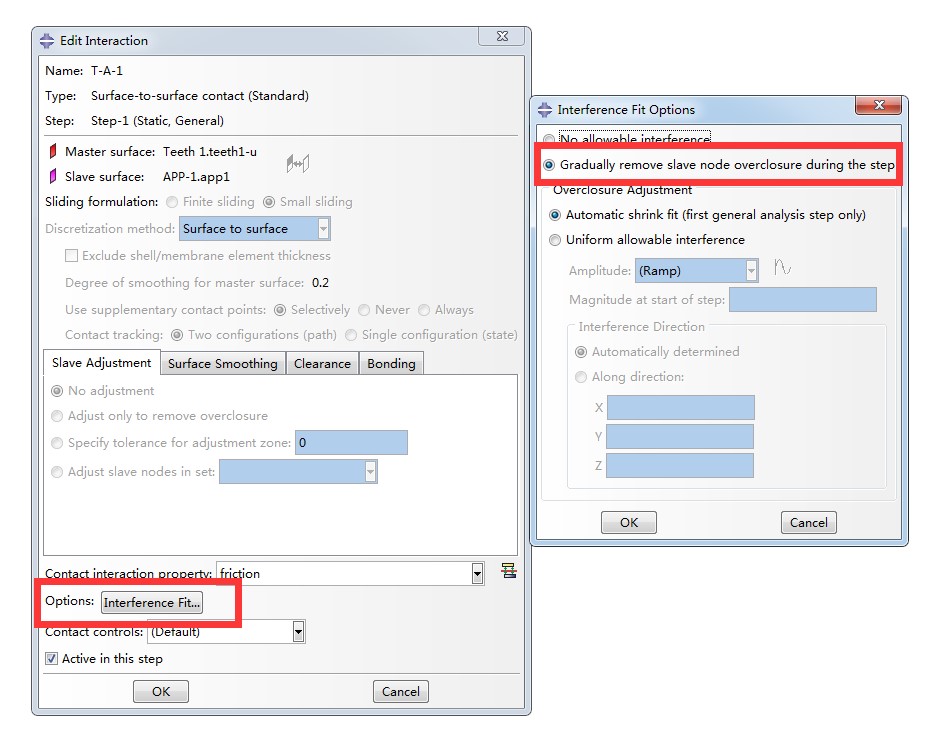
**

**Fig. S2** Interference load setting. In order to resolve the interference between the clear aligner and the crown, the automatic shrink fit algorithm which gradually removed slave node over-closure during the analysis step was applied to resolve the penetration

**
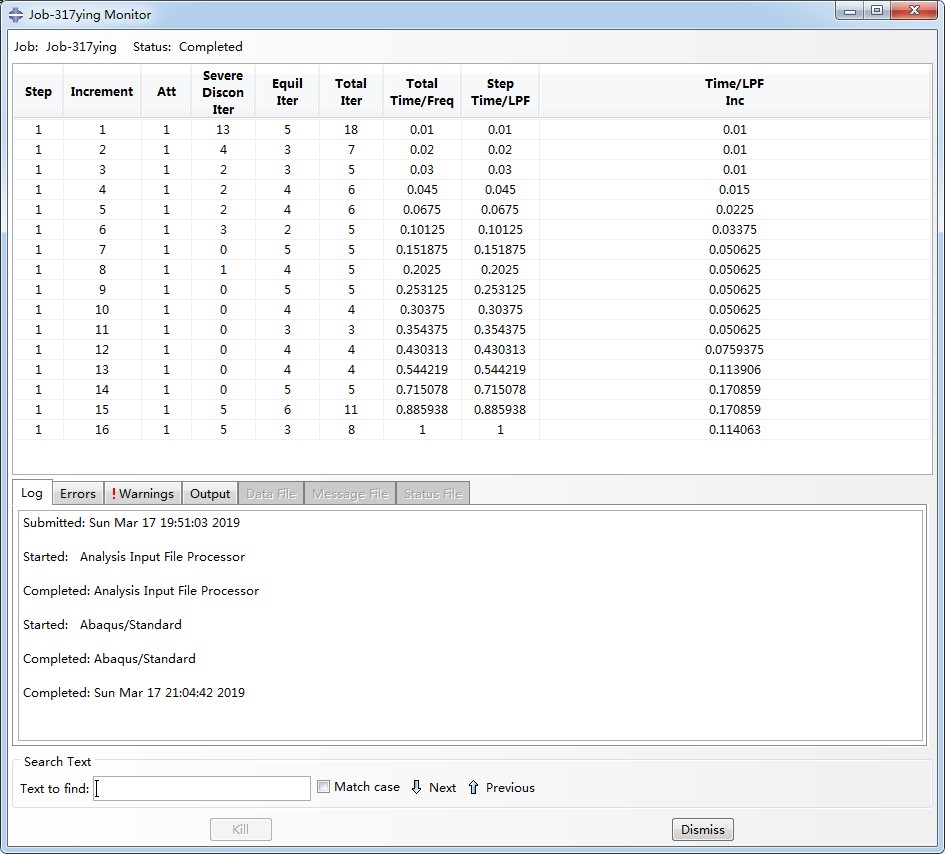
**

**Fig. S3** The iterative calculation process, taking model G0 as an example

**
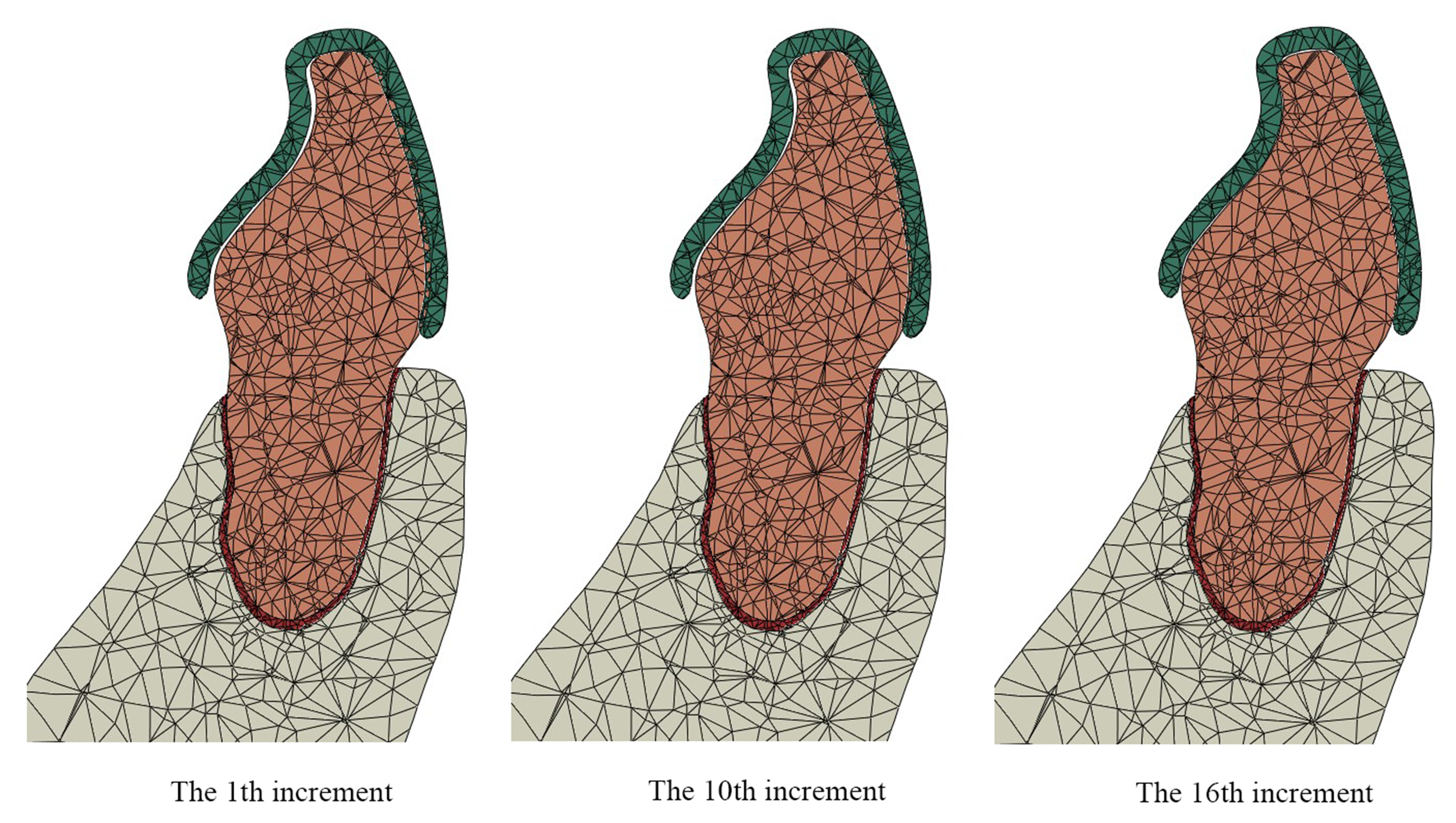
**

**Fig. S4** The change of the relative position between the clear aligner and the crown during the iterative calculation, taking model G0 as an example
